# One-pot synthesis of novel chitosan-salicylaldehyde polymer composites for ammonia sensing

**DOI:** 10.1038/s41598-023-50243-9

**Published:** 2024-01-02

**Authors:** Ahmed Muhammed Saeed, Ahmed Gaber Taha, Hemat Mohamed Dardeer, Moustafa Fawzy Aly

**Affiliations:** 1 Department of Chemistry, Faculty of Science, Al-Azhar University, Cairo, 11884 Egypt; 2 Department of Chemistry, Faculty of Science, South Valley University, Qena, 83523 Egypt

**Keywords:** Environmental sciences, Chemistry, Materials science, Nanoscience and technology

## Abstract

Chitosan (Chs)-salicylaldehyde (Sal) polymer derivatives were formed via the reaction of Chs-Sal with zinc oxide nanoparticles (ZnO NPs) and beta-cyclodextrin (β-CD). These polymers were synthesized through inclusion with β-CD and doping with ZnO NPs to give pseudopolyrotaxane and Chs-Sal/ZnO NPs composite, respectively, for low-temperature detection and sensing of NH_3_ vapors as great significance in environmental control and human health. Additionally, the polymer (Chs-Sal/β-CD/ZnO NPs) was prepared via the insertion of generated composite (Chs-Sal/ZnO NPs) through β-cyclodextrin ring. The structural and morphological characterizations of the synthesized derivatives were confirmed by utilizing FTIR, XRD and, SEM, respectively. Also, the optical properties and thermal gravimetric analysis (TGA) of the synthesized polymers were explored. The obtained results confirmed that using β-CD or ZnO NPs for modification of polymer (Chs-Sal) dramatically enhanced thermal stability and optical features of the synthesized polymers. Investigations on the NH_3_-sensing properties of Chs-Sal/β-CD/ZnO NPs composite were carried out at concentrations down to 10 ppm and good response and recovery times (650 s and 350 s, respectively) at room temperature (RT) and indicated that modification by β-CD and doping with ZnO NPs effectively improves the NH_3_-sensing response of Chs-Sal from 712 to 6192 using Chs-Sal/β-CD/ZnO NPs, respectively, with low LOD and LOQ of 0.12 and 0.4 ppb, respectively.

## Introduction

Polymers have emerged as inevitable in our daily lives because of their many applications in various fields such as medical^1,2^, automobile^3^, aerospace^4^, electronics^5^, packaging^6^, and food^7^. It is well known that synthetic polymers are harmful to the environment due to their non-degradation^8^. Therefore, researchers tend to replace these synthetic polymers with biopolymers such as cellulose, chitin, chitosan and, poly peptides^9–12^. However, chitosan is superior to the other bio-polymers due to its crystallinity, nitrogen richness, hydrophilicity, and high viscosity ionic conductivity. Chitosan is extracted from the shells of shrimp and other easily crustaceans^13^. Chitosan's amino group has drawn a lot of attention since it creates a corresponding Schiff base (SB)^14–17^. Numerous uses for this imino group exist, such as sorbents, corrosion inhibitors, medicinal agents, sensors^18,19^, and important biological reaction intermediates^16,20–23^. Finally, modification of chitosan through chemical, radiation, enzymatic and, physical methods can produce a biopolymer that has more tendencies to interact with other components^24^. Chitosan has also different active groups such as primary and secondary hydroxyl groups in its monomer, so it can be chemically modified by interaction with other components, such as metal ions, and organic compounds^25^.

Cyclodextrin (CD) is well-known type of macrocyclic compound obtained by intermolecular connection of d-(+)-glucopyranosyl units^26^. CDs are one of the oligosaccharides class with an important role in the pharmaceutical research and global market, which are formed by starch under the function of amylase enzyme^27,28^. Primarily, it is separated into three products: α-, β-, and γ-CDs, each consisting of 6, 7, and 8 glucose units. β-CD is typically the most utilized due to its moderate molecular void space and inexpensive production cost, making it more appropriate for a variety of applications^29^.

Interestingly, ammonia (NH_3_) is used in various fields such as food, industrial, cleaning, and chemical manufacturing^30–32^. Moreover, ammonia has also been utilized as a key signal in the detection of conditions including diabetes, renal illness, malignant tumors, and lung cancer since it is a metabolite in human life^33^. Despite its use, NH_3_ is a poisonous gas that is detrimental to human health due to its colorlessness and toxicity^34^. A large dose of ammonia affects negatively on the human body and may cause eye discomfort, sore throat, skin irritation, and respiratory system irritation^35^. Inorganic compounds (metal-oxides) and organic materials (conducting polymer) have been the subject of studies on NH_3_ gas sensors^18,19,36^. The metal-oxides-based gas sensors may have a high sensing response and low detection limit.

ZnO NPs and polymer/ZnO nanocomposites as NH_3_ sensors have been reported by different researches^37–41^. The results demonstrated that the polymer/ZnO nanocomposites exhibited higher response towards NH_3_ with good response and recovery times in addition to low limit of detection (LOD).

It was reported by our previous work that the chitosan–N-acetylisatin/β-CD/ZnO NPs composite was used in bone tissue engineering and afforded good results in this field. So, we expected these types of polymers to have many applications in various fields^42^, especially in toxic gas sensing.

We study the modification of chitosan-salicylaldehyde (Chs-Sal) polymer through inclusion into β-CDs and doping with ZnO nanoparticles (NPs) to give (Chs-Sal/β-CD)pseudopolyrotaxane and Chs-Sal/ZnO NPs composite. Moreover, modify Chs-Sal polymer through insertion into β-CDs and doping with ZnO nanoparticles (NPs) in one pot reaction to afford (Chs-Sal/β-CD/ZnO NPs) pseudopolyrotaxane composite in a cheap, simple and eco-friendly route. The modified polymers exhibit elevated crystallinity, wide optical properties, and thermal stability making them suitable for wide applications such as toxic vapor and gas sensing.

## Materials and methods

### Materials

Chitosan (deacetylation degree above 85%, CAS. No = 9012-76-4), salicylaldehyde (98.5%, CAS. No = 90-02-8), β-CD (CAS. No = 7585-39-9), ZnO NPs (purity over 97%, > 50 nm), glacial acetic acid, and dimethylformamide (DMF) (≥ 99.8%) were supplied from Sigma-Aldrich, Milwaukee, Wisconsin, USA. All chemicals and solvents used are of analytical grade and are used as received without further purification.

### Synthesis of Chs-Sal polymer

**Figure 1:**
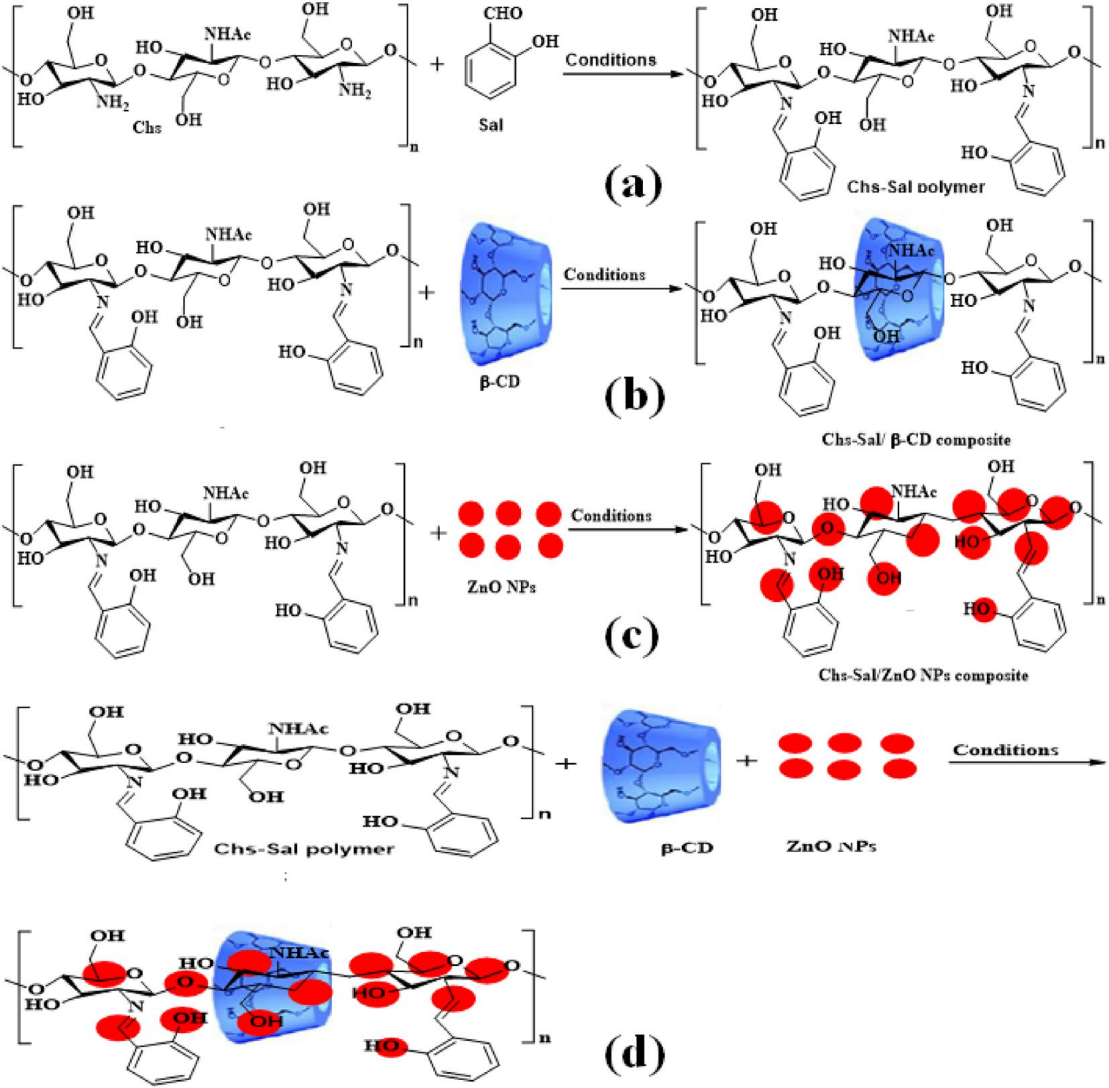
Synthesis of (**a**) Chs-Sal polymer, (**b**) Chs-Sal/β-CD composite, (**c**) of Chs-Sal/ZnO NPs composite, and (**d**) Chs-Sal/β-CD/ZnO NPs composite, respectively.

A solution of salicylaldehyde (0.40 g in 10 ml MeOH) was prepared at room temperature with stirring for 6 h before the temperature was raised to 100 °C for 1 h, according to Fig. 1a. Chitosan powder (1.0 g) in 50 ml of glacial acetic acid (1% v/v) was then added. The Chs-Sal polymer was obtained as a yellow powder by evaporating the solvent at room temperature.

### Synthesis of pseudopolyrotaxane (Chs-Sal/β-CD)

A mixture of Chs-Sal polymer (1.0 g) and β-CD (2.0 g) in DMF (25 ml) was stirred at room temperature for 24 h, Fig. 1b. The obtained precipitate dried at room temperature to afford Chs-Sal/β-CD as a pale-yellow powder.

### Synthesis of Chs-Sal/ZnO NPs composite

A mixture of ZnO NPs (0.10 g) and Chs-Sal polymer (1.0 g) in DMF (25 ml) was agitated for 30 h at room temperature. To obtain green crystals of Chs-Sal/ZnO NPs, the hydrogel that had formed was transferred into a Petri plate and allowed to dry at room temperature (Fig. 1c).

### Synthesis of Chs-Sal/β-CD/ZnO NPs composite

For six hours, a solution containing 1.0 g of Chs-Sal polymer and 0.10 g of ZnO NPs in 25 ml of DMF was stirred at room temperature. Next, β-CD (2.0 g) was added to DMF (25 ml) and stirred while left at room temperature for 48 h (Fig. 1d). The formed hydrogel was poured into a Petri dish and dried at room temperature to afford Chs-Sal/β-CD/ZnO NPs composite as a pale-yellow crystal.

### Characterizations

#### Fourier-transformation infrared (FTIR)

FTIR spectroscopy was used to examine the structure of the generated derivatives at room temperature utilizing potassium bromide disc (KBr) (using an infrared spectrometer, a Jasco Model 4100 from Japan), specifically in the wavenumber range of 4000 to 400 cm^−1^.

#### X-ray diffraction (XRD)

XRD measurements were performed at room temperature using a powder diffractometer (Brucker D8 Advance, Germany) fitted with a Cu K radiation source, yielding values of = 1.5406 and 2 in the range of (5°–80°) for the crystallite size and phase structure of the polymers

#### Scanning electron microscope (SEM)

A 20 kV accelerated voltage SEM (JEOL SEM model JSM-5500—Japan) was utilized to examine the morphological structures of the formed derivatives.

#### Thermogravimetric analysis (TGA)

Using a TGA (SDT Q600 V20.9 Build 20-Germany) with a 5 °C/min heating rate up to 400 °C and a 5 ml/min nitrogen gas flow, the thermal stability of the synthesized derivatives was assessed. A data collecting and handling mechanism is built into the thermal analyzer (TA-50WSI).

#### UV–visible spectroscopy

The optical characteristics of the produced polymers were determined using UV–visible spectroscopy. The UV–visible spectra were obtained by using a UV–visible spectrophotometer (PG Instruments, model T80, UK) and quartz cells with a path length of 1 cm with wavelengths ranging from 200 to 800 nm. To modify the baseline, DMF was utilized as a blank.

### Gas sensing studies

The sensing properties of the synthesized polymer composites were studied by utilizing a homemade system comprises a 5 L glass chamber and a laptop interfaced digital multimeter for signal acquisition^36,43,44^. The sensor was fabricated by painting the composite paste on interdigital electrode interdigitated electrodes (5 cm × 4 cm × 2 cm) and dried at 45 °C for 24 h.

Different volumes of volatile liquids were injected into the bottle using a syringe to obtain the corresponding concentrations. The relation = Ra/Rg, where Ra and Rg stand for the sensor's resistances in the air and in the presence of the tested gas, respectively, was used to calculate the sensor response. The amount of time it takes to get to 90% of the final equilibrium value is defined as the response and recovery times.

## Results and discussion

### SEM analysis

**Figure 2:**
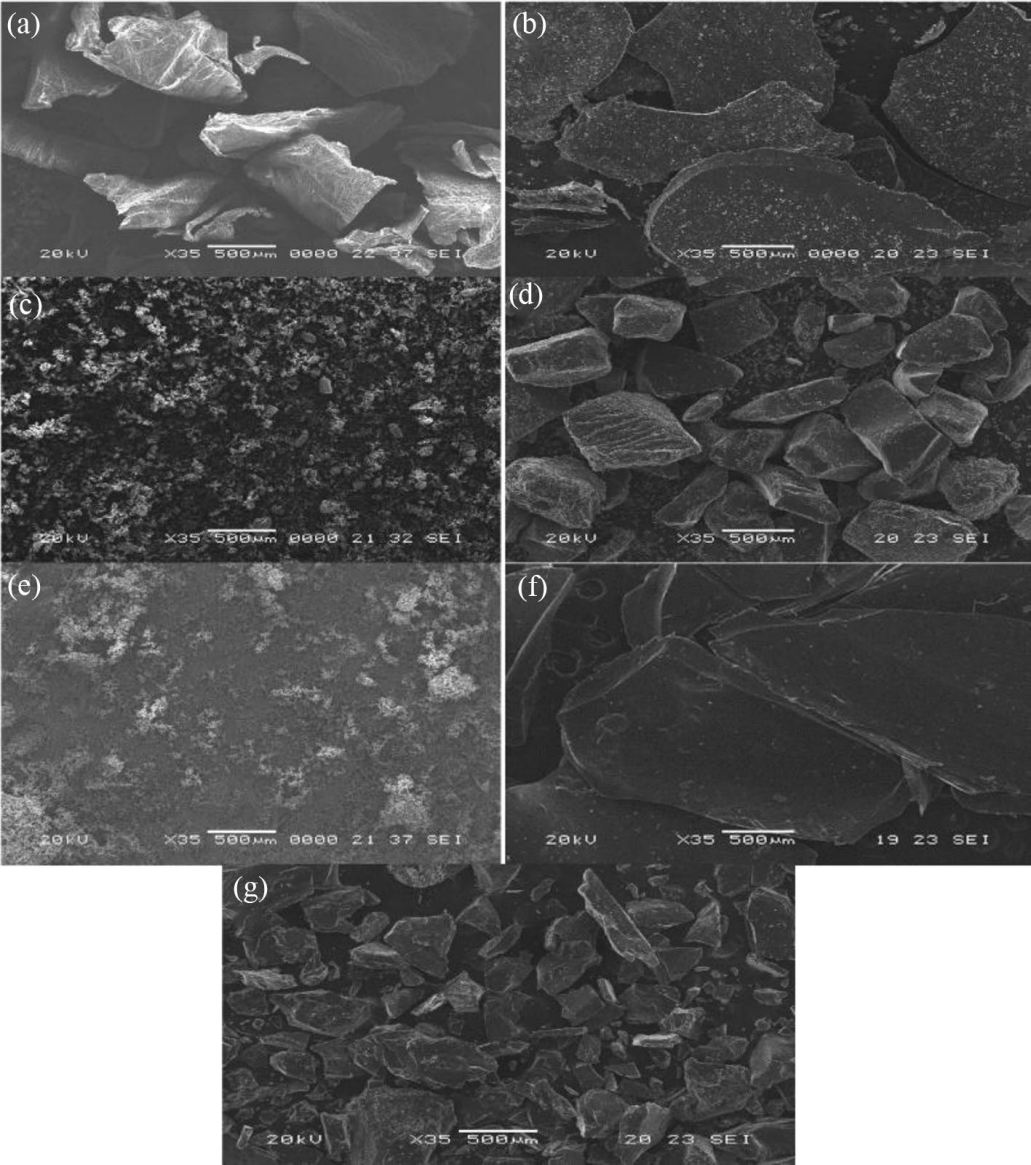
SEM images of (**a**) Chs, (**b**) Chs-Sal, (**c**) β-CD, (**d**) Chs-Sal/β-CD, (**e**) ZnO NPs, (**f**) Chs-Sal/ZnO, and (**g**) Chs-Sal/β-CD/ZnO.

Figure 2 shows the SEM images and the surface morphology of chitosan, β-CD, ZnO NPs, and the generated chitosan polymers (Fig. 2a–g). In contrast to the fibrous nature of the chitosan surface, these images demonstrate the apparent differences between them and the surface appearances that were altered upon reaction. As rough, amorphous slides, the Chs-Sal polymer was visible (Fig. 2b). Additionally, the surface morphology of β-CD and pseudopolyrotaxane polymer showed a high difference between them due to the insertion of Chs-Sal polymer into the β-CD (Fig. 2d). Pseudopolyrotaxane polymer showed a scaly and slightly rough structure with a bigger random particle crystal size. SEM images of Chs-Sal/ZnO NPs composite showed smooth slides without pores surface. The pores of Chs-Sal polymer are considered a particle sink to get ZnO NPs inside it to form the soft surface of the composite (Fig. 2f). The surface morphology of pseudopolyrotaxane polymer was changed in Chs-Sal/β-CD/ZnO NPs composite (Fig. 2g). It has become uneven and aggregated in Chs-Sal/β-CD/ZnO NPs composite.

### XRD analysis

**Figure 3:**
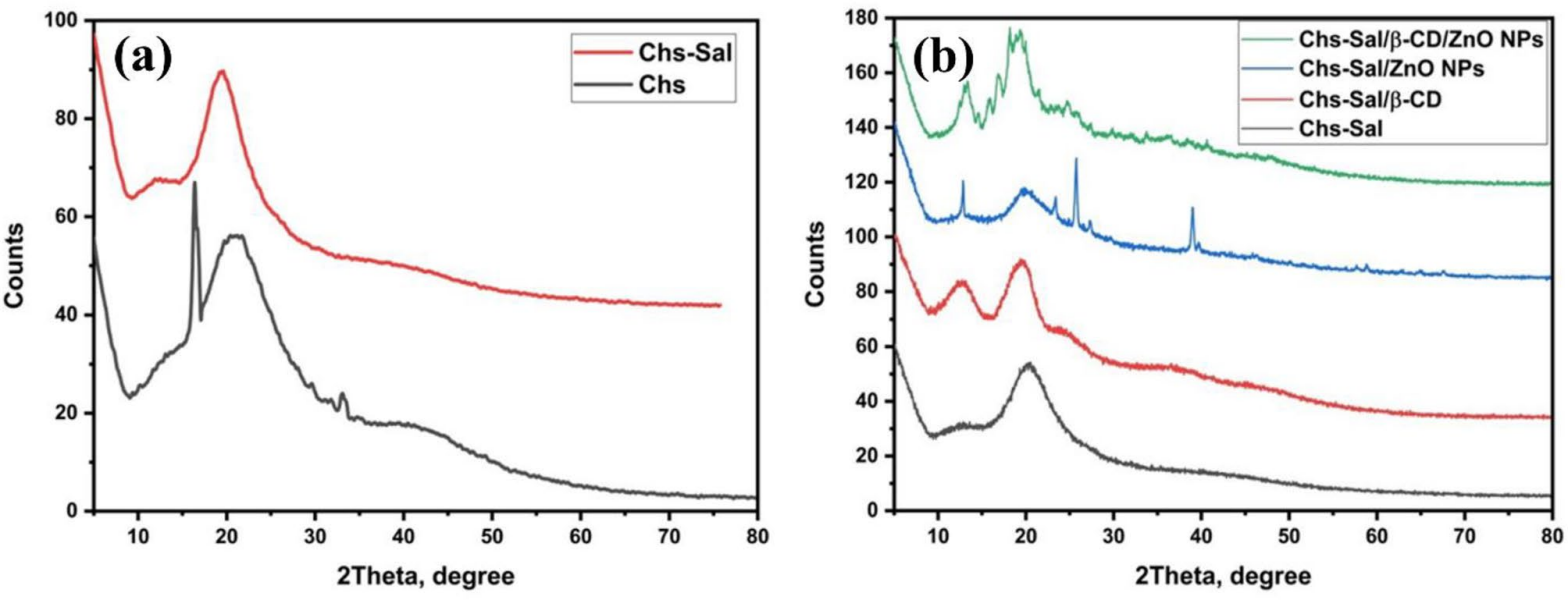
XRD spectra of (**a**) chitosan and chitosan-salicylaldehyde, and (**b**) Chs-Sal, Chs-Sal/β-CD, Chs-Sal/ZnO, and Chs-Sa/β-CD/ZnO.

The phase structure and crystallite size of chitosan (Chs), Chs-Sal polymer, β-CD, pseudopolyrotaxane polymer, ZnO NPs, Chs-Sal/ZnO NPs, Chs-Sal/β-CD/ZnO NPs composite, were accomplished using XRD analysis (Fig. 3a,b) at 25 °C, in the range of 5° to 80°. The diffraction peaks that are characteristic of Chs were observed at 2θ = 8.6° and 20°, indicating that it is semi-crystalline^45–48^. XRD analysis showed the formation of Schiff base (SB) (Chs-Sal polymer) via the changing of amino group nature. Moreover, the difference in the crystal size and crystallinity of Chs was indicated by disappearance peaks in 16.4°, 33.6°, and the appearance of a slight peak in 13.8°. Additionally, the crystallinity values of Chs and the formed Chs-Sal polymer showed 57.7 and 50.6%, respectively. The crystallinity value of Chs-Sal decreased due to the formation of SB and cleavage of hydrogen bonds^49^. The XRD pattern of pseudopolyrotaxane polymer showed a sharp diffraction angle at 14.2° and the crystallinity value was 48.3% and the crystal size increased compared to Chs-Sal polymer (crystal size values of Chs-Sal and pseudopolyrotaxane polymer were 2.4 and 3.2 nm, respectively). Decreased crystallinity in the case of the pseudopolyrotaxane polymer is due to the Chs-Sal chain being inserted into the electron-rich cavity of the cyclodextrin rings. After addition of ZnO NPs to the Chs-Sal polymer gave a different pattern in XRD analysis. In the blind polymer, a broad peak decreased in intensity and some distinct peaks emerged at 15.9°, 17.9°, and 29.9°. Additionally, the cost of the crystallinity value (46.1%) this difference indicates the formation of hydrogen bonding between Chs-Sal and ZnO NPs. Finally, Chs-Sal/β-CD/ZnO NPs composite XRD result showed broad peaks at 13.3°, 13.7°, 14.2°, 14.6°, 17.6°, 18.2°, higher crystal size 5.78 nm and lower crystallinity value 45%. The Scherrer equation was used to calculate the crystal size of the synthesized polymers^50^.

$${\text{D}} = {\text{K}}\lambda {/}\beta {\text{cos}}\theta$$

where *λ* is the wavelength of the X-ray; *β*, the half width of the diffraction peak; *θ*, diffraction angle; and *k*, constant. And the crystallinity values were calculated from this equation:

$$\% {\text{ Crystallinity}} = \left[ {\left( {\text{area under the crystalline peaks}} \right)/\left( {\text{area under all peaks}} \right)} \right] \, \times \, 100.$$

### Fourier-transform infrared spectroscopy (FT-IR)

**Figure 4:**
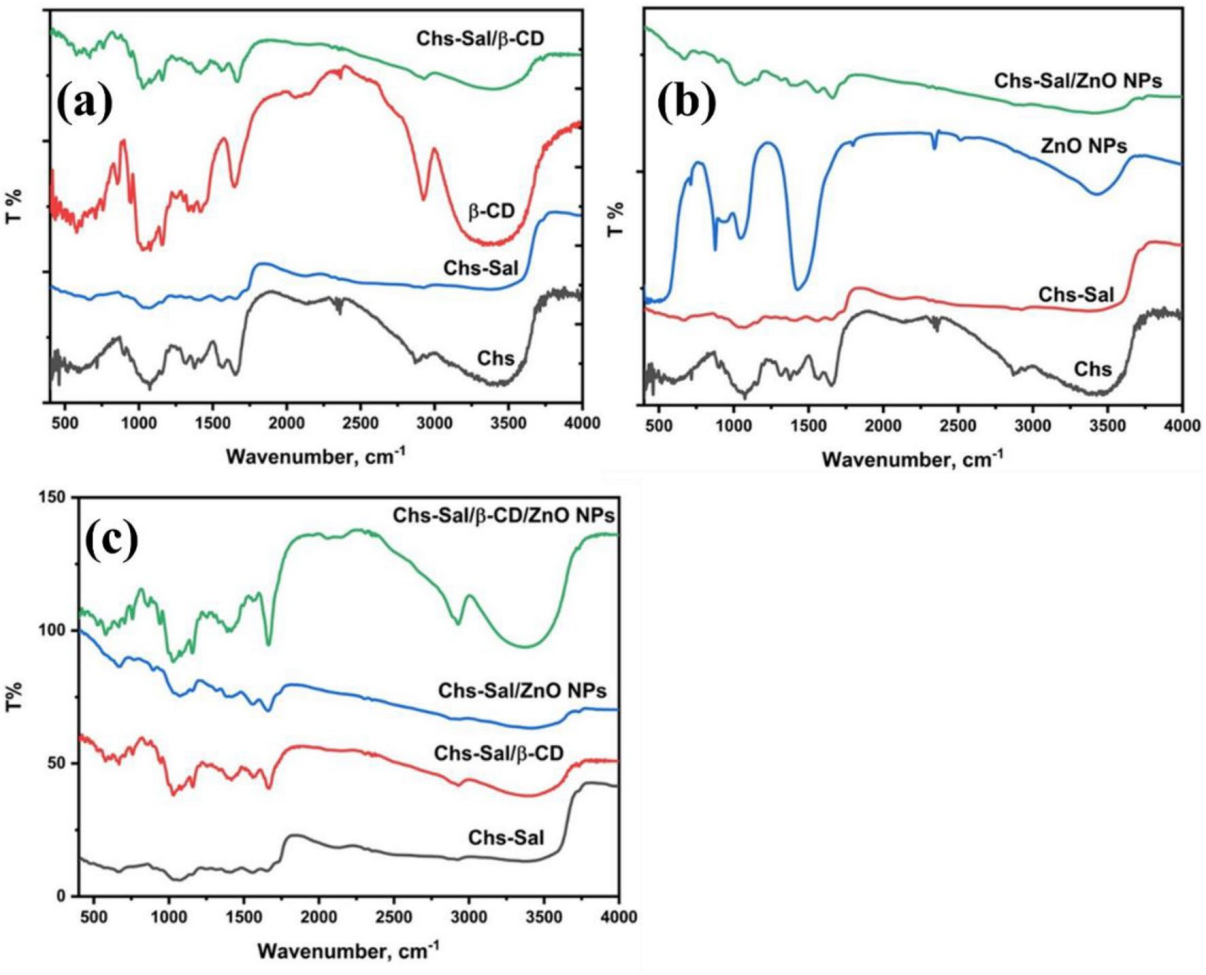
FT-IR spectra of (**a**) Chs, Chs-Sal, and β-CD and Chs-Sal/β-CD, (**b**) Chs, Chs-Sal, ZnO NPs and Chs, and Chs-Sal/ZnO NPs, and (**c**) Chs-Sal, Chs-Sal/β-CD, Chs-Sal/ZnO NPs and Chs-Sal/β-CD/ZnO NPs, respectively.

FT-IR was used to confirm the formation of the components. Figure 4a shows the difference between the Chs and Chs-Sal polymer as a SB. The hydrocarbon bond C–H appeared at 2978 cm^−1^, and the –OH group characteristic peak in Chs appeared at 3334 cm^−1^, superimposed over the N–H stretching band. In addition, the peaks of –C=O amide and –NH_2_ were observed at 1657 cm^−1^ and 1598 cm^−1^. The spectrum of SB polymer showed C=N band at 1631 cm^−1^ which confirmed the Chs-Sal polymer formation. Additionally, C–C bonds stretching and bending vibrations on the aromatic ring of the aldehyde and the stretching vibration of –C–O of Sal appeared at 1490, 820, and 1250 cm^−1^, respectively^51–53^. There is no detectable residual of free Sal, as evidenced by the absence of the salicylaldehyde characteristic band in the area 1660–1730 cm^−1^ in the SB spectrum. Table 1 provides a summary of the differences between the absorbance bands of chitosan and Chs-Sal polymer.

**Table 1:** FT-IR changes of pure Chs and Chs-Sal polymer.

Functional groups	Wave number, cm^−1^		
	Chs	Chs-Sal	∆ν
ν[OH, NH] symmetric	3343	3373	− 30
ν[CH aliphatic]	2978	2960	+ 18
ν[C–O]	1657	1649	+ 8
ν[C–N]	–	1631	–
ν[CH_2_–OH]	1378	1370	+ 7
ν[C–O–C]	1068	1072	− 4

ν=ν (Chs-Sal polymer) − ν(Chs).

It was noticed that the absorption band of the hydroxyl group is slightly high and less intensity than the β-CD sample (Fig. 4a). Also, the ν[OH] symmetric stretching was shifted to a higher frequency and ν[CH-aliphatic] was shifted to a lower frequency compared to those in β-CD. Furthermore, the ν[C–O–C] and ν[CH_2_-O] bending vibrations were shifted to lower frequencies at 1027 and 1158 cm^−1^, respectively. These results confirmed the formation of pseudopolyrotaxane polymer through the reaction of Chs-Sal polymer with β-CD. The increase in frequency is due to the Chs-Sal chain being inserted into the electron-rich cavity of the cyclodextrin rings, which accounts for the observation^54^. Conversely, the drop in frequency can be attributed to the formation of hydrogen bonds and Vander Waals forces between the β-CDs and the hydroxyl groups of Chs and Sal molecules, as well as between β-CDs themselves (Fig. 4a). The absorbance bands of pure β-CD, Chs-Sal polymer, and pseudopolyrotaxane polymer are shown to vary in Table 2.

**Table 2:** FT-IR changes of β-CD, Chs-Sal polymer, and pseudopolyrotaxane polymer**.**

Functional groups	Wave number, cm^−1^				
	∆ν_1_	Chs-Sal polymer	Pseudopolyrotaxane	β-CD	∆ν_2_
ν[OH, NH] symmetric	+ 23	3373	3396	3389	+ 7
ν[CH aliphatic]	− 60	2960	2900	2925	− 25
ν[C–O]	+ 19	1649	1668	–	–
ν[CH_2_–OH]	− 22	1370	1158	1159	− 1
ν[C–O–C]	− 45	1072	1027	1028	− 1

∆ν_1_ = ν(pseudopolyrotaxane) − ν(Chs-Sal polymer), ∆ν_2_ = ν(pseudopolyrotaxane) − ν(β-CD).

The FT-IR spectrum of the Chs-Sal/ZnO NPs polymer (Fig. 4b), showed a broad absorption band at 3408 cm^−1^ corresponding to the stretching vibrations of hydroxyl (OH) groups. The absorption band at 2945 cm^−1^ is attributed to symmetric stretching of aliphatic C–H groups of Chs in polymer blend^14^, which is markedly shifted and decreased in intensity (2930 cm^−1^) upon doping of ZnO NPs. The absorption band at 1660 cm^−1^ is assigned to free C=O stretching vibration^55^. The absorption bands at 1560 and 1413 cm^−1^ corresponding to the polymer (C=N) group bending^56^ and stretching vibration of (CH_2_–OH), were shifted towards higher wave numbers and decreased in their intensities, because of the interaction between the polymer blend chains and the ZnO NPs^57^. The band at 1073 cm^−1^ which is attributed to C–O–C stretching became less intense and was shifted to lower wavenumber. Table 3 summarizes the variations in absorption bands between Chs-Sal polymer and Chs-Sal polymer doped with ZnO NPs.

**Table 3:** FT-IR changes of Chs-Sal polymer, Chs-Sal/ZnO NPs composite and ZnO NPs.

Functional groups	Wave number, cm^−1^				
	∆ν_1_	Chs-Sal polymer	Chs-Sal/ZnO NPs	ZnO NPs	∆ν_2_
ν[OH, NH] symmetric	+ 107	3373	3480	3333	+ 147
ν[CH aliphatic]	− 30	2960	2930	–	–
ν[C–O]	− 11	1649	1660	–	–
ν[NH-bending]	+ 50	1570	1620	1553	+ 67
ν[CH_2_–OH]	+ 43	1370	1413	1393	+ 20
ν[C–O–C]	+ 1	1072	1073	1090	− 13

∆ν_1_ = ν(Chs-Sal/ZnO NPs) − ν(Chs-Sal polymer), ∆ν_2_ = ν(Chs-Sal/ZnO NPs) − ν(ZnO NPs).

These changes (shift and decreased intensity) indicated the strong interaction between these functional groups in the polymer blend and ZnO NPs. The absorption band at 675 cm^−1^ appeared due to the stretching mode of the amide groups attached to ZnO NPs^58,59^ (Fig. 4b).

Figure 4c shows the difference between Chs-Sal polymer, Chs-Sal/ZnO NPs composite, Chs-Sal/β-CD, and Chs-Sal/β-CD/ZnO NPs composite. The FT-IR spectrum of the Chs-Sal/β-CD/ZnO NPs composite showed a broad absorption band of OH group at 3375 cm^−1^ with increasing intensity compared to the last polymers. The absorption band of aliphatic C–H groups at 2927 cm^−1^, was markedly shifted and increased in intensity. The absorption band at 1663 cm^−1^ was assigned to free C=O stretching vibration, and C–N group appeared at 1663 cm^−1^ with high intensity. The absorption bands at 1158 and 1028 cm^−1^ correspond to the stretching vibration of (CH_2_–OH) group and C–O–C stretching became more intense and shifted to a lower wavenumber. The strong interaction between these functional groups in the polymer blend of pseudopolyrotaxane and ZnO NPs is indicated by these changes (shift and increase in intensity). The absorption band at 756 cm^−1^ appeared due to the stretching mode of the amide groups attached to ZnO NPs. Table 4 summarizes the differences between the absorption bands of the Chs-Sal/ZnO NPs polymer, Chs-Sal/β-CD polymer, and Chs-Sal/β-CD/ZnO NPs polymer.

**Table 4:** FT-IR changes of Chs-Sal/ZnO NPs polymer, Chs-Sal/β-CD polymer and Chs-Sal/β-CD/ZnO NPs polymer.

Functional groups	Wave number, cm^−1^				
	∆ν_1_	Pseudopolyrotaxane	Chs-Sal/β-CD/ZnO NPs	Chs-Sal/ZnO NPs	∆ν_2_
ν[OH, NH] symmetric	− 21	3396	3375	3480	− 105
ν[CH aliphatic]	+ 27	2900	2927	2930	− 3
ν[C–O]	− 5	1668	1663	1660	+ 3
ν[CH_2_–OH]	− 2	1158	1156	1413	− 257
ν[C–O–C]	+ 1	1027	1028	1073	− 45

**∆**ν_1_ = ν(Chs-Sal/β-CD/ZnO NPs) − ν(pseudopolyrotaxane), ∆ν2 = ν(Chs-Sal/β-CD/ZnO NPs) − ν(Chs-Sal/ZnO NPs).

### Optical properties

**Figure 5:**
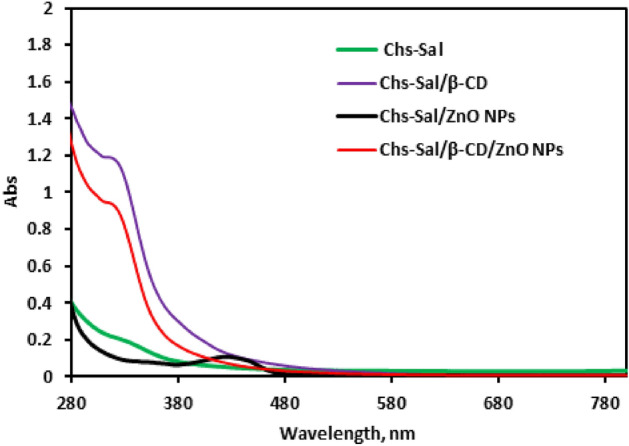
UV spectra of Chs-Sal, Chs-Sal/β-CD, Chs-Sal/ZnO NPs and Chs-Sal/β-CD/ZnO NPs.

UV–visible spectroscopy of Chs-Sal polymer, Chs-Sal/ZnO NPs composite, Chs-Sal/β-CD, and Chs-Sal/β-CD/ZnO NPs composite were recorded in the range of 200–800 nm at 25 °C (Fig. 5). The UV–visible spectrum of Chs-Sal polymer showed a broad peak at the region 327–360 nm due to the presence of π bond (–N=CH–). It's interesting to note that adding ZnO NPs and β-CD to the Chs-Sal polymer caused the characteristic peak to slightly shift blue, from 360 to 340 and 356 nm, respectively. Also, in the case of the addition of β-CD and ZnO NPs into Chs-Sal as a one pot-reaction gave a sharp peak in 439 nm. These data approved the formation of Chs-Sal/ZnO NPs, Chs-Sal/β-CD, and Chs-Sal/β-CD/ZnO NPs composite. Additionally, the energy gap E*g* of the prepared polymers was calculated depending on the UV–visible absorption spectra according to Tauc's formula. The values of E*g* for Chs-Sal, Chs-Sal/β-CD, Chs-Sal/ZnO NPs composite and Chs-Sal/β-CD/ZnO NPs composite were 4.4, 3.55, 2.65, and 3.11 eV, respectively. The lower E*g* of Chs-Sal/β-CD/ZnO NPs composite (3.11 eV) compared to Chs-Sal (4.4 eV) makes the resistance of the sensor more likely to change in the presence of gas due to electron transition which leads to gas sensitivity improvement^37,60–63^.

### Thermal analysis

**Figure 6:**
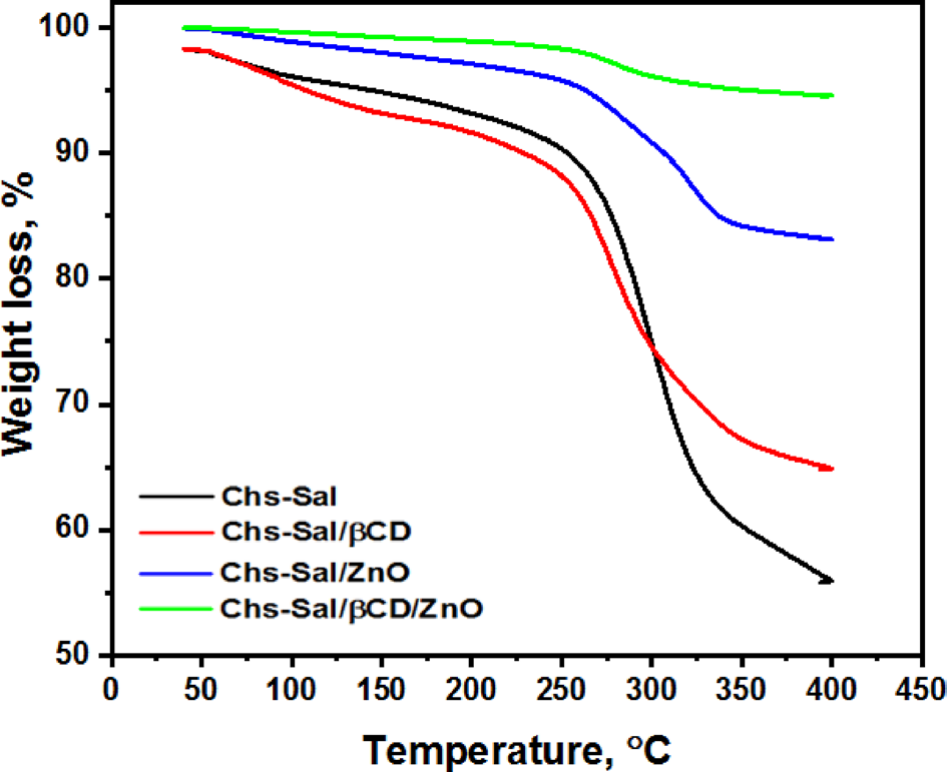
TGA spectra of Chs-Sal, Chs-Sal/β-CD, Chs-Sal/ZnO NPs and Chs-Sal/β-CD/ZnO NPs.

The thermal stability and heat resistance of the synthesized polymers were investigated using thermogravimetric analysis (TGA). TGA curves for Chs-Sal, Chs-Sal/ZnO NPs composite, Chs-Sal/β-CD, and Chs-Sal/β-CD/ZnO NPs composite are displayed in Fig. 6. Figure 6 clearly shows that, overall, the thermal stability of the Chs-Sal/β-CD composite was lower than that of the Chs-Sal polymer, while the weight loss at 350 °C was 40% and 34%, respectively. In the case of Chs-Sal was an organic polymer directly exposed to heat. But when the polymer was inserted into β-CD, it was preserved from the heat more and the loss of weight was less. The heat resistance of Chs-Sal/ZnO composite was improved by the incorporation of ZnO NPs because of the strong bond between zinc and oxygen in ZnO NPs, whereas the weight loss of Chs-Sal/ZnO at 350 °C was 17%. Conversely, when compared to Chs-Sal/β-CD, the thermal stability of the ZnO NPs composite increased by 55% at the same temperature. This was because the polymer's stability was increased by the addition of ZnO NPs. After addition of ZnO NPs into Chs-Sal/β-CD gave a more stable polymer, whereas the weight loss of Chs-Sal/β-CD/ZnO at 350 °C was 5%. The order of thermal stability for the four samples is based on the results shown in Fig. 6 and was as follow: Chs-Sal/β-CD/ZnO NPs ˃ Chs-Sal/ZnO NPs ˃ Chs-Sal/β-CD ˃ Chs-Sal.

### Gas sensing properties

**Figure 7:**
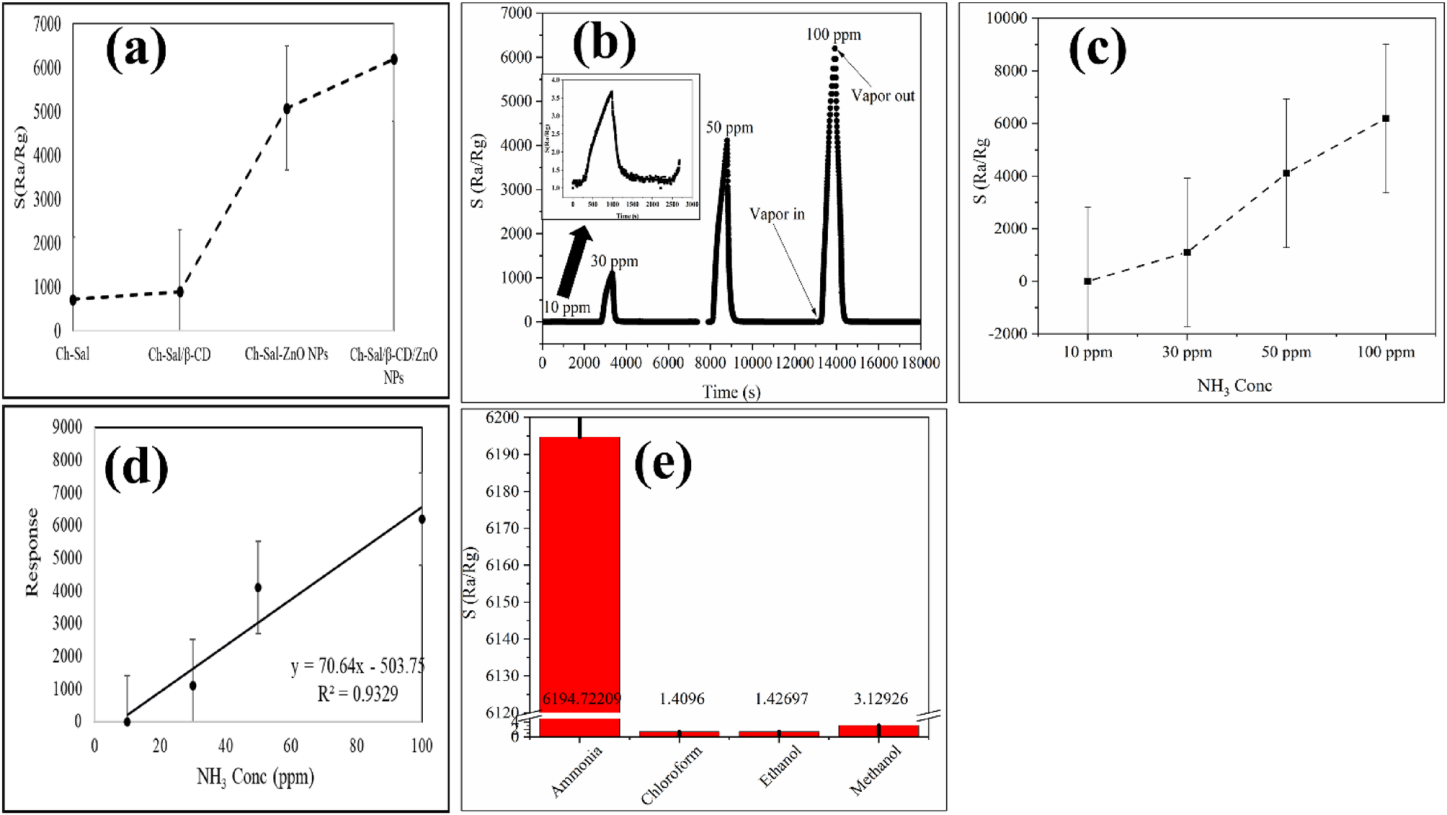
Maximum response comparison of Chs-Sal, Chs-Sal/β-CD, Chs-Sal/ZnO NPs and Chs-Sal/β-CD/ZnO NPs towards NH_3_ (100 ppm) at room temperature (**a**), gas responses of Chs-Sal/β-CD/ZnO NPs towards different concentrations of NH_3_ (**b**), maximum response of Chs-Sal/β-CD/ZnO NPs vs different concentrations of NH_3_ (**c**), linear fitting of Chs-Sal/β-CD/ZnO NPs response towards different NH_3_ concentrations (**d**), and selective response of Chs-Sal/β-CD/ZnO NPs to some gases (100 ppm) at room temperature (**e**), respectively.

Figure 7b,c show the response of the polymer composite as a sensor towards various concentrations of NH_3_ at RT. The Chs-Sal/β-CD/ZnO NPs composite sensor exhibited high sensing characteristics toward NH_3_ down to 10 ppm concentration. It was observed that the sensor response was increased by increasing NH_3_ concentration and recovered after purging the chamber with air, indicating reversible response characteristics of the composite (Fig. 7b). The sensor response and recovery times were 650 s and 350 s, respectively, indicating that Chs-Sal/β-CD/ZnO NPs composite is suitable for gas sensing applications. Besides, the selectivity of the Chs-Sal/β-CD/ZnO NPs composite sensor has also been studied at room temperature upon exposure to various 100 ppm gases/vapors (NH_3_, chloroform, ethanol, and methanol) as depicted in Fig. 7e. The results indicate that the Chs-Sal/β-CD/ZnO NPs composite sensor exhibits the highest response when exposed to 100 ppm NH_3_ which may to be due to its highest electron-donating ability compared to the other analytes^64^. Furthermore, the selective response of the composite toward NH_3_ might be related to ZnO (transition metal oxide) which has more than one oxidation state and d^10^ electronic configuration that could retain cations with its filled orbital and be reduced when it reacted with reducing analyte like NH_3_ to generate free electrons at RT^65^. Another reason for the high selectivity towards NH_3_ may be due to the lone pair of electrons on the N atom, which are more likely to occupy the vacant orbital on Zn^2+^, leading to the formation of a stronger binding energy^37^, suggesting that the Chs-Sal/β-CD/ZnO NPs composite has excellent selectivity in NH_3_-monitoring application. Figure 7a shows the response comparison of Chs-Sal, Chs-Sal/β-CD, Chs-Sal/ZnO NPs, and Chs-Sal/β-CD/ZnO NPs composite as sensors to 100 ppm of NH_3_ at RT. The obtained observations indicated that Chs-Sal/β-CD/ZnO NPs composite exhibited improved NH_3_ sensing response compared to other polymers (712 for Chs-Sal, and 6192 for Chs-Sal/β-CD/ZnO NPs, respectively). The limit of detection (LOD) is used as an important parameter for representing the sensing properties of the sensors, a high sensitivity usually has a low limit of detection. The calculations were achieved using the variation in the gas response at baseline (without analyte gas) using the root-mean-square deviation (rms). The rms deviation was calculated on 50 points at baseline^66^ using RMS _noise_ = $\sqrt{\sum_{i}{(Si-S)}^{2}/N}$, where S_i_ is the experimental data point, S is the corresponding value calculated from the curve-fitting, and N is the number of data points, respectively. The LOD is calculated as 3(RMS_noise_/slope) (according to the IUPAC definition), where the slope is obtained from linear fitting of the response vs concentration (Fig. 7d). LOD of the prepared composite Chs-Sal/β-CD/ZnO NPs was 0.12 ppb surpassing the threshold value for NH_3_ gas indicated by the National Institute for Occupational Safety and Health (NIOSH) (total weight average (TWA) permissible ammonia exposure limit is 25 ppm and the short-term exposure limit (ST) (for 15 min) is 35 ppm)^67^. Furthermore, this LOD is less than other NH_3_ sensors such as 0.4 ppm^64^, 11 ppm^68^, and 0.78 ppb^69^, respectively. The limit of quantification (LOQ) was calculated using 10(RMS_noise_/slope)^70^ and was 0.4 ppb which is lower than that reported in other studies^71–73^.

Gas sensing behavior of the semiconductor is based on the strong interaction between the analyte gas molecules and the adsorbed oxygen species at the surface of the sensing material. Initially, the adsorbed oxygen molecules attract electrons from the ZnO NPs surface because they have high electron affinity^74^, so, it is believed that the sensing mechanism occurs at the surface of the ZnO NPs. It was reported that defects such as Zn interstitials and oxygen vacancies that are found on ZnO NPs surface enable oxygen molecules in the ambient atmosphere to be adsorbed easily due to the differences in chemical potential energy^75^. When the Chs-Sal/β-CD/ZnO NPs composite is exposed to atmospheric air, the oxygen molecules trap the electrons from the conduction band of ZnO NPs and chemisorbed on the surface forming O^2−^, O^−^ and O^2−^ by increasing depletion layer width. This leads to increasing the surface resistance of the sensor. Upon injecting ammonia, the adsorbed oxygen species react with the NH_3_ molecules forming H_2_O, N_2_, and three electrons which are injected into the conduction band of ZnO NPs by decreasing the depletion layer width and decreasing sensor resistance as a result. Upon the recovery of the sensor, the adsorbed ammonia molecules get desorbed from the ZnO NPs surface and increase the depletion layer width again and the resistance of the sensor is increased and returns to the initial baseline value^38^. The mechanism of the process is expressed by the following equations:

1 $${\text{O}}_{2} \;({\text{gas}}) \, \to {\text{ O}}_{2} \;({\text{ads}})$$

2 $${\text{O}}_{2} \;({\text{ads}}) \, + {\text{ e}}^{ - } \;({\text{from}}\;{\text{ZnO}}) \, \to {\text{ O}}_{2}^{ - } \;({\text{ads}})$$

3 $${\text{O}}^{2 - } \;({\text{ads}}) + {\text{e}}^{ - } ({\text{from}}\;{\text{ZnO}}) \to {\text{O}}^{ - } \;({\text{ads}})$$

4 $${\text{O}}^{ - } \;({\text{ads}}) + {\text{e}}^{ - } \;({\text{from}}\;{\text{ ZnO}}) \to {\text{O}}^{ - 2} \;({\text{ads}})$$

5 $$2{\text{NH}}_{3} + 3{\text{O}}^{ - } \;({\text{ads}}) \to {\text{N}}_{2} + 3{\text{H}}_{2} {\text{O }} + 3{\text{e}}^{ - } \;({\text{to }}\;{\text{ZnO}})$$

In addition, The presence of OH groups of β-CD may also participate in the adsorption of NH_3_ molecules through hydrogen bonding (hydrophilic outer side), and thus provides a favorable environment and more active sites for the adsorption of more NH_3_ molecules^76–80^. Moreover, many vapor channels in the cavities of β-CD in addition to the inclusion of Ch-Sal-ZnO into these cavities may enhance the sensor response of Chs-Sal/β-CD/ZnO NPs composite^81^. Also, to further improve the sensitivity, it is necessary to introduce a conductive substrate such as ZnO NPs to increase the specific surface area of the composite^77^. Furthermore, the inner cavity of β-CD may occupied by traces of water vapor from the atmospheric air which may also interact with NH_3_ vapor^82,83^, leading to higher response value in the Chs-Sal/β-CD/ZnO NPs composite.

## Conclusion

Stirring at room temperature of Chs polymer as an amine with Sal as a carbonyl component afforded Chs-Sal polymer. The obtained Chs-Sal was added to β-CD to form pseudopolyrotaxane inclusion complex and doped with ZnO NPs to produce the corresponding composite in a one-pot, adequate, simple, cost-effective, and eco-friendly environmentally method. Chs, Sal, ZnO NPs, and β-CD were reacted as one pot reaction to form Chs-Sal/β-CD/ZnO NPs composite polymer. These derivatives were confirmed by FT-IR and XRD analyses, while SEM analysis indicated remarkable morphological changes between them. UV–visible results confirmed the improvement of the optical properties of the prepared samples, as well as the decreasing of E*g* of Chs-Sal/β-CD/ZnO NPs composite (3.11 eV) compared to Chs-Sal (4.4 eV) which enhances the sensitivity. The thermal stability of Chs-Sal polymer was enhanced by doping with ZnO NPs. Gas sensing results showed that the modification of chitosan-salicylaldehyde polymer through inclusion into β-CDs and doping with ZnO nanoparticles (NPs) showed high sensitivity (from 712 for Chs-Sal to 6192 for Chs-Sal/β-CD/ZnO NPs), good response and recovery times (650 s and 350 s, respectively) and low LOD and LOQ of 0.12 and 0.4 ppb, respectively, in addition to high selectivity towards NH_3_ vapors which is related to its highest electron-donating ability compared to the other analytes, making them have a potential application prospect in NH_3_ monitoring.

## Data Availability

All data generated or analyzed during this study are included in this published article.
